# Mitral valve surgery for ischemic papillary muscle rupture: outcomes from the Japan cardiovascular surgery database

**DOI:** 10.1007/s11748-020-01418-y

**Published:** 2020-06-25

**Authors:** Tomoyuki Fujita, Hiroyuki Yamamoto, Junjiro Kobayashi, Satsuki Fukushima, Hiroaki Miyata, Kizuku Yamashita, Noboru Motomura

**Affiliations:** 1 Department of Cardiothoracic Surgery, National Cerebral and Cardiovascular Center, 6-1 Kishibeshimmachi, Suita, Osaka, 564-8565 Japan; 2 Department of Healthcare Quality Assessment, Graduate School of Medicine, The University of Tokyo, Tokyo, Japan; 3 Japan Cardiovascular Surgery Database Organization, Tokyo, Japan

**Keywords:** Acute myocardial infarction, Acute mitral regurgitation, Papillary muscle rupture, Mitral valve replacement, Mitral valve repair

## Abstract

**Background:**

Ischemic papillary muscle rupture (PMR) is a catastrophic complication following acute myocardial infarction (AMI). We evaluated early outcomes of PMR by using data from the Japan Cardiovascular Surgery Database, a nationwide Japanese registry.

**Methods:**

We retrospectively analyzed data from 196 patients diagnosed with PMR following AMI in Japan between January 2014 and December 2017. Risk factors for operative mortality and severe complications following mitral valve surgery were analyzed.

**Results:**

The 30-day and hospital mortality rates were 20% and 26%, respectively. Chronic hemodialysis, abrupt rupture after AMI, resuscitation before surgery, and preoperative venoarterial extracorporeal membrane oxygenation were associated with mortality. Mitral valve replacement was chosen mainly (90%) for surgical correction of mitral regurgitation in these patients. There was no significant difference in short-term outcomes between mitral valve replacement versus mitral valve repair, despite non-matched characteristics in background between the treatment groups. Concomitant coronary artery bypass grafting had no impact on short-term outcomes.

**Conclusions:**

Information derived from the nationwide database of patients with AMI-associated PMR show that PMR is a rare condition in the modern era. However, PMR is a severe disease with a mortality rate as high as 26%. The severity of the condition is associated with the risk for poor outcomes.

**Electronic supplementary material:**

The online version of this article (10.1007/s11748-020-01418-y) contains supplementary material, which is available to authorized users.

## Introduction

Ischemic papillary muscle rupture (PMR), left ventricular rupture, and ventricular septal rupture are life-threatening mechanical complications following acute myocardial infarction (AMI) [1–6]. Ischemic PMR causes acute mitral regurgitation (MR) and results in cardiogenic shock, while acute elevation of left ventricular end-diastolic pressure produces pulmonary edema [1–6]. Vasopressors and inotropic agents are necessary to elevate systemic pressure, but these drugs in combination with increased preload can worsen pulmonary edema. Prompt surgical correction of MR is crucial. However, surgery in patients with AMI, especially emergency surgery, is always challenging. Mechanical support, such as intra-aortic balloon pumping (IABP) and venoarterial extracorporeal membrane oxygenation (VA-ECMO), is important to bridge to surgery for further hemodynamic support, although no data support a reduction in mortality rates with these therapies [7]. The percutaneous left ventricular assist device has recently been reported to be a useful bridge to surgery [8]. Mitral valve surgery to repair or replace the valve is the gold-standard treatment for this condition, as recommended in current guidelines [9, 10]. However, reported mortality rates after mitral valve surgery are as high as 13–55% [1–6]. Because of the rarity of ischemic PMR, few reports have been published on the subject. The risk factors for ischemic PMR remain unclear and the choice of mitral valve replacement (MVR) versus mitral valve repair (MVr) is controversial [5, 6, 11]. Therefore, we launched this study to explore early outcomes and risk factors for mitral valve surgery for PMR by using the Japan Cardiovascular Surgery Database (JCVSD).

## Methods

### JCVSD

The JCVSD is a national database used to assess surgical outcomes after cardiovascular procedures on a multicenter basis throughout Japan. The JCVSD captures clinical information from 99% of Japanese hospitals that perform cardiovascular surgery and comprised 591 hospitals in 2018 [12, 13]. The JCVSD is now part of the National Clinical Database, which includes information concerning not only cardiovascular surgery but also thoracic surgery, general surgery, and neurosurgery. The data registration project was approved by the institutional review board at each participating hospital; review boards approved the collection of their data for use in the JCVSD. The method of data collection from the JCVSD has been previously described [12, 13]. More than 300 variables in the data collection form are nearly identical to those of the STS National Database. The content of the JCVSD is available online at https://www.jacvsd.umin.jp and can be verified with reference to that of the STS National Database (https://sts.org). The data manager of each participating hospital was responsible for forwarding patient data electronically to the central office. The rate of data entry was monitored annually in the central office to ensure comprehensive input of the data. The accuracy of submitted data was maintained through a data audit; administrative office members and investigators who had previously used the JCVSD for clinical studies randomly visited a participating hospital every month.

### Patients

For the present study, use of data from 2014 to 2017 was approved by the Data Utilization Committee of the JCVSD. Following JCVSD approval in 2018, data analysis was undertaken. Ischemic PMR resulting from AMI was identified in 196 patients during the study period. Data collection, analysis, and reporting were approved by the National Cerebral and Cardiovascular Center Institutional Review Board on 17 October 2018 (IRB No.: M30-092).

### Study endpoints

The primary endpoint of this study was 30-day mortality and operative mortality, defined as death within 30 days after mitral surgery and death during the primary hospitalization, respectively. Major morbidities during postoperative hospitalization included stroke, prolonged mechanical ventilation for 24 h or more, atrial fibrillation, newly required hemodialysis, including continuous veno-veno hemodialysis (or filtration), pneumonia, and deep sternal wound infection. We also compared the backgrounds and outcomes of patients who underwent MVR versus MVr.

### Definition of urgency of surgery

Urgency of surgery was expressed as follows, according to the definition in the JCVSD website. Emergent: Patients requiring emergency operations in which there should be no delay in providing operative intervention. Salvage: Patients undergoing cardiopulmonary resuscitation en route to the operating room or during induction of anesthesia. Urgent: patients requiring urgent surgery within 24 h in order to minimize the chance of further clinical deterioration. Elective: none of the above.

### Statistical analysis

Statistical analysis was performed with STATA 16 (STATA Corp., College Station, TX, USA). Continuous variables are presented as median (interquartile range). Categorical variables are presented as *n* (%). A 2-tailed *P* < 0.05 was considered significant. Univariate comparisons of categorical and continuous variables were made with Fisher’s exact test and Wilcoxon rank-sum test, respectively. Univariate logistic regression analysis was performed to identify risk factors for operative mortality and/or stroke. Risk factors included age category (10-year age groups), sex, preoperative chronic dialysis, ST-elevated or non-elevated myocardial infarction, duration from onset to surgery, history of percutaneous coronary intervention (PCI), history of resuscitation, preoperative VA-ECMO, MVR or MVr, and concomitant coronary artery bypass grafting (CABG).

## Results

Patient characteristics are shown in Table 1. The median patient age was 74 years; 26% of the patients were older than 80 years. Thirty-nine percent of patients were women. The median body mass index was 22 kg/m^2^; only 5% of patients had a body mass index above 30 kg/m^2^. Hypertension and dyslipidemia were found in 55% and 31% of patients, respectively. Diabetes mellitus was found in 25% of patients. Renal dysfunction [estimated glomerular filtration rate (eGFR) less than 60 mL/min/1.73 m^2^] was present in 79% of patients; 2.6% of these were receiving dialysis. In terms of type of AMI, 69% of patients were diagnosed with ST-elevated myocardial infarction and 19% were diagnosed with non-ST-elevated myocardial infarction. One-third of the patients developed PMR within 24 h after AMI. As for the severity of disease, 90% of patients were NYHA class 3 or 4, 70% had cardiogenic shock, 32% required VA-ECMO, and 80% required IABP support. Furthermore, 12% of patients required resuscitation before surgery. Coronary angiogram was performed in 89% of patients and showed significant stenosis (75% or more) in the left anterior descending coronary artery (43%), circumflex artery (52%), and right coronary artery (47%). The remaining patients underwent surgery without coronary angiogram.

**Table 1:** Baseline characteristics of patients with papillary muscle rupture

Variables	Total
Number	196
age	74 (67–80)
Age category	
− 59	14 (7.1%)
60–69	51 (26.0%)
70–79	80 (40.8%)
80 −	51 (26.0%)
Sex (male)	119 (60.7%)
BMI (median)	22.25 (20.5–24.55)
BMI (> 30)	10 (5.1%)
Hypertension	107 (54.6%)
Dyslipidemia	60 (30.6%)
Diabetes mellitus	48 (24.5%)
Respiratory disease	25 (12.8%)
Peripheral vascular disease	4 (2.0%)
Cerebral infarction	19 (9.7%)
eGFR	40.1 (28.1–54.9)
eGFR (< 60)	153 (78.9%)
Chronic hemodialysis	5 (2.6%)
Previous cardiac surgery	3 (1.5%)
Type of MI	
STEMI	136 (69.4%)
NSTEMI	38 (19.4%)
Unknown	22 (11.2%)
Interval between MI to PMR	
~ 24 h	67 (34.2%)
24 h ~	116 (59.2%)
Unknown	13 (6.6%)
LVEF < 30%	20 (10.2%)
NYHA class3or4	177 (90.3%)
Cardiogenic shock	140 (71.4%)
Resuscitation within 1 h before surgery	23 (11.7%)
Preoperative VA-ECMO	63 (32.1%)
Preoperative IABP	159 (81.1%)
Coronary angiogram performed	174 (88.8%)
Coronary lesion	
LMT	5 (2.9%)
LAD	75 (43.1%)
LCx	90 (51.7%)
RCA	82 (47.1%)

*BMI* body mass index, *eGFR* estimated glomerular filtration rate, *IABP* intra-aortic balloon pump, *LAD* left anterior descending artery, *LCx* left circumflex artery, *LMT* left main trunk, *LVEF* left ventricular ejection fraction, *MI* myocardial infarction, *NSTEMI* non-ST-elevation myocardial infarction, *NYHA* New York Heart Association, *PMR* papillary muscle rupture, *RCA* right coronary artery, *STEMI* ST-elevation myocardial infarction, *VA-ECMO* venoarterial extracorporeal membrane oxygenation

A summary of interventions and surgeries is shown in Table 2. Emergency mitral valve surgery was performed in 57% of patients; only 13% of patients underwent elective surgery. PCI and concomitant CABG were performed in 47% and 31% of patients, respectively. Nearly 90% of patients underwent MVR and only 10% underwent MVr.

**Table 2:** Characteristics of Surgeries and Interventions

Variables	Total
Number	196
Urgency of surgery	
Emergent	112 (57.1%)
Urgent	49 (25.0%)
Salvage	9 (4.6%)
Elective	26 (13.3%)
PCI performed within this episode	93 (47.4%)
Concomitant CABG	60 (30.6%)
MVR	176 (89.8%)
Procedure time (min)	289 (240.5–366)
Cardiopulmonary bypass time (min)	156 (123–204)
Cardiac arrest time (min)	92 (75–121)

*CABG* coronary artery bypass grafting, *MVR* mitral valve replacement, *PCI* percutaneous coronary intervention

The 30-day and hospital mortality rates were 20% and 26%, respectively (Table 3). The incidence of the composite outcome of mortality and stroke was 32%. Prolonged ventilation was needed in 28% of patients and new-onset hemodialysis was needed in 18%. The median postoperative stay was 28 days. During hospitalization, 11% of patients developed pneumonia and 3% developed deep sternal infection.

**Table 3:** Outcomes

Variables	Total
Number	196
30-day mortality	40 (20.4%)
Operative mortality	50 (25.5%)
Stroke	16 (8.2%)
Composite of death or stroke	62 (31.6%)
Prolonged ventilation (> 24hrs)	54 (27.6%)
Atrial fibrillation	38 (19.4%)
Newly dialysis	35 (17.9%)
Pneumonia	22 (11.2%)
Deep sternal infection	6 (3.1%)
Postoperative stay	28 (17–57)

To analyze the risk factors for 30-day and hospital mortality and for the composite outcome of mortality and stroke, several background, and surgical variables were selected (Table 4). The odds ratio increased with age, although the difference was not statistically significant. Chronic hemodialysis, resuscitation within 1 h before surgery, and preoperative VA-ECMO were risk factors for 30-day mortality, hospital mortality, and the composite outcome of mortality and stroke. An interval longer than 24 h between AMI and PMR was protective for mortality. There were no significant differences in short-term outcomes between patients who underwent concomitant CABG versus those who did not or between those who underwent MVR versus MVr.

**Table 4:** Predictors of 30-day mortality, hospital mortality, and composite outcome of mortality and stroke: univariate analysis

Variables	30-day mortality		Operative mortality		Operative mortality or storke	
	Odds ratio (95% CI)	*p* value	Odds ratio (95% CI)	*p* value	Odds ratio (95% CI)	*p* value
Age category						
− 59	1.00 (1.00–1.00)		1.00 (1.00–1.00)		1.00 (1.00–1.00)	
60–69	1.73 (0.19–15.72)	0.62	1.12 (0.21–5.97)	0.90	2.27 (0.45–11.45)	0.32
70–79	3.77 (0.46–30.84)	0.22	2.42 (0.50–11.68)	0.27	3.23 (0.67–15.47)	0.14
80−	5.42 (0.65–45.18)	0.12	3.00 (0.60–14.95)	0.18	3.27 (0.66–16.26)	0.15
Sex (male)	0.58 (0.29–1.16)	0.12	0.55 (0.29–1.06)	0.07	0.70 (0.38–1.29)	0.25
Chronic hemodialysis	6.24 (1.01–38.72)	0.05	12.61 (1.37–115.66)	0.03	9.17 (1.00–83.86)	0.05
STEMI	1.02 (0.42–2.45)	0.97	0.97 (0.43–2.20)	0.94	1.00 (0.33–3.12)	0.98
Interval between MI to PMR (> 24hrs)	0.30 (0.14–0.64)	< 0.01	0.39 (0.20–0.78)	0.01	0.60 (0.32–1.14)	0.12
Resuscitation within 1 h before surgery	3.67 (1.47–9.14)	0.01	2.56 (1.04–6.27)	0.04	2.68 (1.11–6.48)	0.03
Preoperative VA-ECMO	6.92 (3.24–14.79)	< 0.01	5.14 (2.59–10.20)	< 0.01	6.15 (3.16–11.94)	< 0.01
Cocomitant CABG	0.83 (0.38–1.79)	0.63	0.85 (0.42–1.72)	0.64	0.90 (0.46–1.73)	0.74
MVR	2.48 (0.55–11.16)	0.24	1.42 (0.45–4.45)	0.55	1.09 (0.40–2.98)	0.87

*CABG* coronary artery bypass grafting, *MI* myocardial infarction, *PMR* papillary muscle rupture, *STEMI* ST-elevation myocardial infarction, *VA-ECMO* venoarterial extracorporeal membrane oxygenation

Differences in backgrounds of patients who underwent MVR versus MVr were evaluated. The MVr group was younger and had higher eGFR than the MVR group and included fewer shock patients (Table 5). MVr was less often selected in emergency situations. The operation time, cardiopulmonary bypass time, and cardiac arrest time were similar in both groups, and concomitant CABG was performed equally in both groups. Surgical outcomes, including mortality and stroke, were similar in both groups.

**Table 5:** Comparison of characteristics and outcomes of patients with MVr versus MVR

Variables	MVr	MVR	p-value
Number	20	176	
Patient background			
Age	66.5 (62–76)	75 (68–81)	0.003
Sex (male)	17 (85.0%)	102 (58.0%)	0.027
eGFR	49.65 (41.95–61.8)	38.85 (26.4–54)	0.020
Interval between MI to PMR			
~ 24 h	1 (5.0%)	66 (37.5%)	0.004
24 h ~	18 (90.0%)	98 (55.7%)	
Unknown	1 (5.0%)	12 (6.8%)	
Cardiogenic shock	7 (35.0%)	133 (75.6%)	< 0.001
Resuscitation within 1 h before surgery	0 (0.0%)	23 (13.1%)	0.14
Preoperative VA-ECMO	4 (20.0%)	59 (33.5%)	0.31
Preoperative IABP	11 (55.0%)	148 (84.1%)	0.004
Intervention and surgery			
Urgency of surgery			
Emergent	3 (15.0%)	109 (61.9%)	< 0.001
Urgent	8 (40.0%)	41 (23.3%)	
Salvage	0 (0.0%)	9 (5.1%)	
Elective	9 (45.0%)	17 (9.7%)	
Cocomitant CABG	6 (30.0%)	54 (30.7%)	1.00
Procedure time (min)	286 (238.5–386)	290 (240.5–365.5)	0.95
Cardiopulmonary bypass time (min)	166.5 (129–209.5)	153 (122–204)	0.30
Cardiac arrest time (min)	105 (85.5–144.5)	92 (75–120)	0.12
Outcomes			
30-day mortality	2 (10.0%)	38 (21.6%)	0.38
Hospital mortality	4 (20.0%)	46 (26.1%)	0.79
Stroke	2 (10.0%)	14 (8.0%)	0.67
Composite of death or stroke	6 (30.0%)	56 (31.8%)	1.00
Prolonged ventilation (> 24hrs)	4 (20.0%)	50 (28.4%)	0.84
Atrial fibrillation	3 (15.0%)	35 (19.9%)	0.77
Newly dialysis	4 (20.0%)	31 (17.6%)	0.76
Pneumonia	2 (10.0%)	20 (11.4%)	1.00
Deep sternal infection	0 (0.0%)	6 (3.4%)	1.00
Postoperative hospital stay	25 (19–52)	28.5 (16.5–57)	0.84

*CABG* coronary artery bypass grafting, *eGFR* estimated glomerular filtration rate, *IABP* intra-aortic balloon pump, *MI* myocardial infarction, *MVr* mitral valve repair, *MVR* mitral valve replacement, *PMR* papillary muscle rupture, *VA-ECMO* venoarterial extracorporeal membrane oxygenation

## Discussion

PMR is a very rare condition, especially in the modern era. The APEX-AMI trial, which recruited patients with ST-elevation myocardial infarction from 17 countries and 296 sites from 2004 to 2006, found a 0.26% incidence of PMR following AMI; more recently that rate was 0.029%, according to data derived from the National Inpatient Sample in the USA from 2005 to 2014 [3, 4]. Our study included data from 196 patients in the JCVSD, which captured clinical information from 99% of Japanese hospitals that performed cardiovascular surgery from 2014 to 2017 (4 years). According to data from the Japanese Circulation Society (https://www.j-circ.or.jp), which captures clinical information given from the 62% of the hospitals which have cardiology departments, the estimated number of AMI patients was 288,922 during the same period in Japan. Therefore, the incidence of PMR following AMI in Japan is also very low. Compared with data from past decades, the modern approach to AMI, which includes early reperfusion with thrombolysis or PCI, has successfully decreased the incidence of PMR [4, 14].

**Fig. 1:**
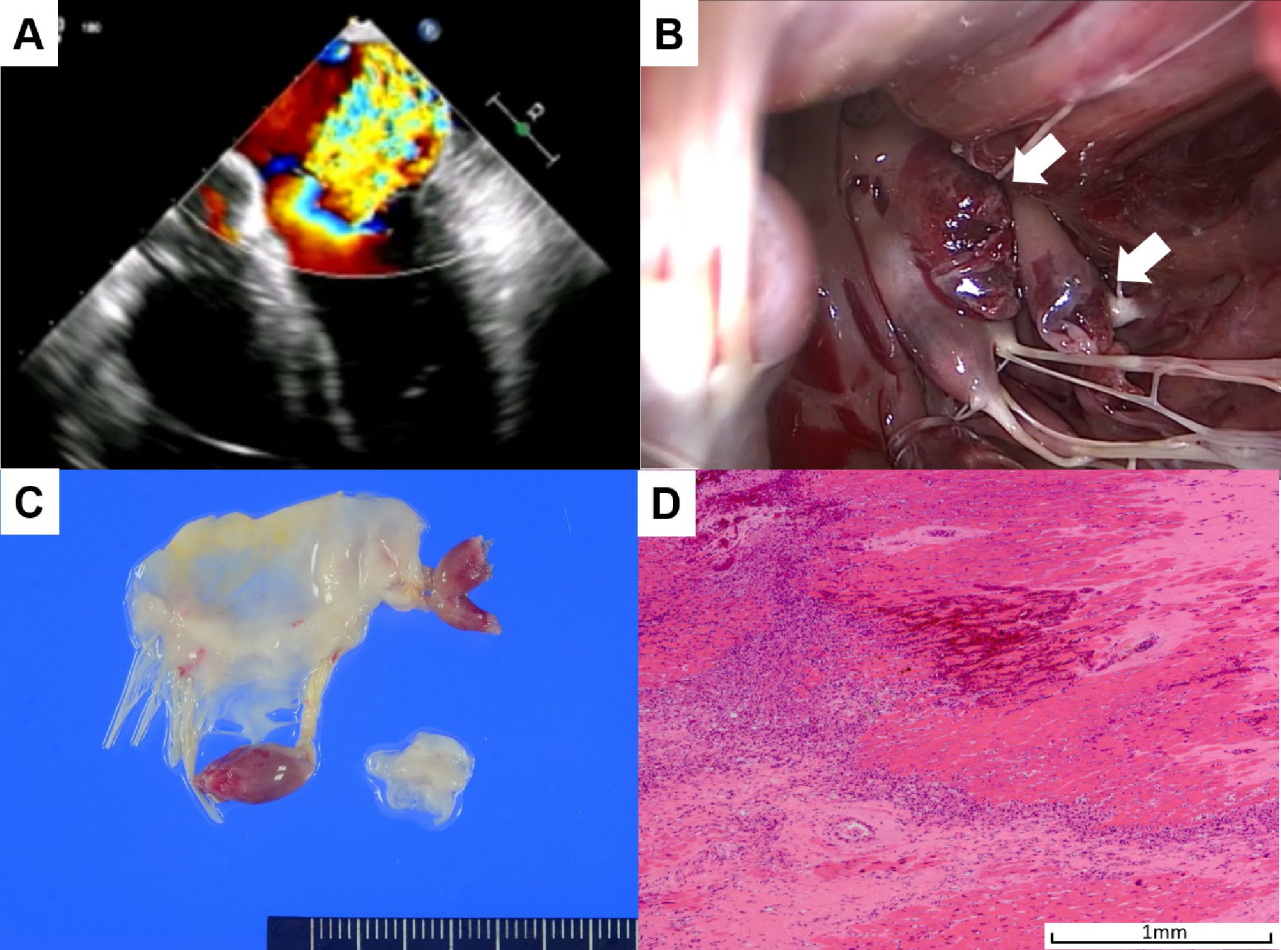
**a** Transesophageal color-flow Doppler image demonstrating severe mitral regurgitation resulting from flail papillary muscle. **b** Intraoperative photograph showing necrotic left ventricular wall and ruptured papillary muscle (white arrows). **c** Surgical specimen of anterior mitral leaflet and ruptured papillary muscle. **d** Pathological examination (HE stain) of papillary muscle showing coagulation necrosis of myocytes

Rupture of the posteromedial papillary muscle occurs 6–12 times more frequently than rupture of the anterolateral papillary muscle. [1] Whereas the anterolateral papillary muscle has a dual blood supply from the left anterior descending and circumflex arteries, the posteromedial papillary muscle has a single blood supply from the posterior descending artery [15, 16]. Complete rupture of the papillary muscles results in catastrophic hemodynamics, as revealed by transesophageal echocardiography with color-flow Doppler (Fig. 1a). Surgical findings include a necrotic left ventricular wall and ruptured papillary muscle (Fig. 1b, c and supplementary file of surgical video). Pathological examination of the papillary muscle shows coagulation necrosis of myocytes (Fig. 1d). These findings indicate that PMR is related to the extent of AMI.

Although PMR is usually diagnosed between 2 to 7 days after AMI, the reported median time to PMR is 13 h [17]. In the present study, 34% of patients presented within 24 h after AMI. At presentation, more than 90% of patients were in severe heart failure (NYHA class 3 or higher) and more than 70% were in cardiogenic shock. Prompt mechanical support, such as IABP (81%) and VA-ECMO (32%), was used as a bridge to surgery. IABP decreases afterload, resulting in less MR and more forward flow from the left ventricle [18]. VA-ECMO, which stabilizes hemodynamics and improves oxygenation, is ideal mechanical support, but can sometimes worsen pulmonary congestion by elevating left ventricular end-diastolic pressure [19]. The findings in the present study that the use of VA-ECMO, the interval between AMI and PMR, and the need for preoperative resuscitation were significant risk factors for mortality suggest that the severity of disease influences the outcomes.

Coronary revascularization is a key to improving short-term and long-term survival rates. Concomitant CABG improved short- and long-term outcomes in recent studies, although there was no significant difference in mortality rates between patients with versus without CABG in this study [5, 6]. Only 31% of patients in the present study underwent concomitant CABG, whereas 47% underwent perioperative PCI, because PCI is generally performed before surgery in current practice. The increasing use of prompt reperfusion therapies for AMI as a class I treatment appears to reduce the necessity of concomitant CABG [4]. Preoperative PCI may contribute to reduce cardiopulmonary bypass time and operation time. The hybrid therapy of PCI and surgery for PMR may be the standard, in the era of primary PCI.

Whereas MVr is reportedly safe and effective, Russo et al. found no significant difference in 5-year survival between MVR and MVr after PMR [5, 11]. In the present study, short-term outcomes were the same for MVR and MVr after PMR. Patients who underwent MVr had lower urgency and required less mechanical support than those who underwent MVR. MVr was performed for only 10% of patients in this study. The procedure time, cardiopulmonary bypass time, and cardiac arrest time were the same in both groups. Long-term follow-up is necessary to find the difference between MVr and MVR for PMR. Concomitant CABG should be considered if catheter revascularization has not been performed.

Recently, analysis of the outcomes of mitral valve surgery for PMR from STS database was published [20]. They analyzed 1342 patients during 8 years in the USA. Similar to our results, 52% of patients required emergent or salvage operation (61.7% in our data), but only 3.1% were connected ECMO (32.1% in our data). The operative mortality was 20% (25.5% in our data) and stroke rate was 5.2% (8.2% in our data). As showing that preoperative VA-ECMO was a risk factor for operative mortality, management to escape from VA-ECMO may be required. Prior PCI was done in 44.5% and concomitant CABG was done in 59.3% of patients in their study, as those were done in 47.4% and 30.6% in our study. Regarding selection of mitral valve surgery in their study, 80% of patients underwent MVR instead of MVr and had severer background such as cardiogenic shock (MVR vs MVr; 65.9% vs 19.3%), ST-Elevation MI (20.4% vs 1.9%), requiring emergency operation or salvage (60.9% vs 17.1%). Although operative mortality was different between MVR and MVr, MVr has been reserved only for select cases in less decompensated patients, who may have potentially partial PMR. Despite some differences were seen between their study in the USA and our study, complexity of managing PMR was exposed similarly.

There are several important study limitations resulting from the nature of the JCVSD. Because post-hospitalization (longitudinal) data were not available, there are no long-term outcome data. Therefore, long-term survival could not be evaluated. Neither the details of PCI (such as timing and lesions treated) nor sites of AMI (anterior, posterior, or inferior) were available in registry data. It is difficult to precisely assess the relationships between comorbidities and in-hospital events. In addition, the timing and sequence of certain clinical events during hospitalization cannot be assessed with accuracy. The JCVSD does not contain details of imaging, laboratory results, or hemodynamic data.

In conclusion, data on patients with AMI-associated PMR collected from the nationwide database show that PMR is a rare condition in the modern era. However, PMR is a severe disease with a mortality rate as high as 26% and associated morbidities, such as kidney injury, pneumonia, and stroke. The severity of the condition, indicated by rapid progression of PMR or cardiogenic shock requiring resuscitation and/or VA-ECMO, is a risk factor for survival. Although revascularization is a key to treatment, aggressive PCI masks the benefit of concomitant CABG in modern Japan. MVR is more selected than MVr for the surgical correction of MR.

## Electronic supplementary material

Below is the link to the electronic supplementary material.

Supplementary file: Surgical video showing mitral valve replacement for papillary muscle rupture (MP4 3975 kb)
